# University Students’ Basic Psychological Needs, Motivation, and Vitality Before and During COVID-19: A Self-Determination Theory Approach

**DOI:** 10.3389/fpsyg.2021.775804

**Published:** 2021-11-25

**Authors:** Florian H. Müller, Almut E. Thomas, Matteo Carmignola, Ann-Kathrin Dittrich, Alexander Eckes, Nadine Großmann, Daniela Martinek, Matthias Wilde, Sonja Bieg

**Affiliations:** ^1^Institute of Instruction and School Development, University of Klagenfurt, Klagenfurt, Austria; ^2^Institute of Pedagogy of Elementary and Primary Education, University College Carinthia, Klagenfurt, Austria; ^3^School of Education, University of Salzburg, Salzburg, Austria; ^4^Faculty of Teacher Education, University of Innsbruck, Innsbruck, Austria; ^5^Faculty of Biology, Bielefeld University, Bielefeld, Germany; ^6^University College Salzburg, Salzburg, Austria; ^7^Department of Educational Psychology, University College Weingarten, Weingarten, Germany

**Keywords:** basic psychological needs, motivation, vitality, university students, COVID-19, distance learning

## Abstract

Self-determination theory assumes that the basic psychological needs for autonomy, competence, and relatedness are associated with motivational regulation. As these basic psychological needs may have been affected by the shift to distance learning, students’ motivational regulation and vitality may have suffered as well. The purpose of this study was to examine the motivational regulation, satisfaction, or frustration of the basic psychological needs and vitality of university students before and after the transition to forced distance learning during the COVID-19 pandemic: Two student samples from Austria and Germany were studied: One was surveyed before the conversion to distance learning (*N*=1,139) and the other at the beginning of forced distance learning (*N*=1,835). The instruments used were the Scales for the Measurement of Motivational Regulation for Learning in University Students (SMR-L), the German version of the Basic Psychological Needs Satisfaction and Frustration Scale, a scale developed by the authors to differentiate the assessment of social relatedness, and the German version of the Subjective Vitality Scale. The results show that the satisfaction of basic psychological needs was significantly lower and the frustration thereof substantially higher during the distance learning period than before the pandemic. Intrinsic motivation and identified regulation were significantly lower during the forced distance learning period, and more controlled forms of motivation were higher than before the pandemic. Structural equation models showed that 42% of the students’ vitality can be explained by motivational regulation and the satisfaction and frustration of their basic needs. Motivational regulation styles functioned (differentiated according to the degree of autonomy) as mediating variables between basic needs and vitality. In terms of theoretical implications, the distinction between approach and avoidance components of introjected regulation was shown to be adequate and necessary, as they explain the outcome vitality differently. The support and avoidance of frustration of basic psychological needs should be considered in distance learning to promote the quality of motivation and students’ vitality.

## Introduction

The COVID-19 pandemic necessitated a paradigm shift in terms of how we interact with each other. It also affected the higher education system. Starting in March 2020, most countries and over 90% of all students from around the world had to partially or completely switch to distance learning, especially during lockdown phases (United Nations Educational Scientific and Cultural Organization: UNESCO, 2020). The sudden and forced change to distance learning may have affected university students’ learning as well as their motivational and psychological well-being. Initial studies show, for example, that rates of depression among students increased (Kaparounaki et al. 2020), whereas the quality of life and emotional well-being decreased, thus decisively changing significant prerequisites and conditions for learning processes (Adnan and Anwar, 2020; Bojović et al., 2020). Learners reported motivational and attentional problems, a lack of direct social interaction, stress, suboptimal support from teachers, and, in some cases, an increase in autonomy that enabled students to self-direct their learning (Händel et al., 2020; Kedraka and Kaltsidis, 2020; Wong, 2020). The few studies to date have consistently concluded that the lack of interaction with peers and instructors is a key problem (e.g., Händel et al., 2020), which has not only motivational and emotional impact, but also affects the success of learning (e.g., Richardson et al., 2015; Holzer et al., 2021).

The extent to which these abrupt changes (have) affected the motivation and psychological well-being of students in higher education compared to before the pandemic is largely an open question. It can be assumed that their motivation and vitality were decisively influenced by mandatory distance learning because of the lack of social interaction and feedback that promotes learning. Feedback goes hand in hand with the development of competence, which is associated with intrinsic motivation, especially in forced distance learning (Hartnett, 2015; Holzer et al., 2021). Moreover, research has shown that promoting or restricting autonomy undermines intrinsic motivation regardless of the learning setting (Ryan and Deci, 2017). The extent to which students in distance learning perceive themselves as autonomous still needs to be determined.

Based on the assumptions of Self-Determination Theory (SDT) (Ryan and Deci, 2017), this study investigated university students’ basic psychological needs satisfaction (BPNS) and needs frustration (BPNF), their motivational regulation, and their vitality during forced distance learning and compares it with data that were gathered before the pandemic. The study examined the extent to which BPNS and BPNF can contribute toward explaining motivational regulation styles and vitality.

## Self-Determination Theory

Motivation is generally understood as “any internal process that energizes, directs, and sustains behavior” (Reeve, 2016, p. 31–32). Or more briefly according to Baumeister (2016): “Wanting change”—wanting to effect change in the self or change in the environment. Accordingly, motivation theories ask about the why of actions, the quality of processes, and the outcomes of motivated behavior. With regard to motivation in learning, it can be assumed that learning motivation refers to the structures and processes that explain the genesis and effects of learning or of a learning action (see Schiefele, 1974).

If we take a closer look at theories of learning motivation, we find many approaches describing the genesis of learning motivation as a function of individual and environmental aspects. The respective focus, however, strongly varies. *Trait theories* underline personality traits which are more or less stable over situations and which regulate the learning processes. *Cognitive theories of action* have provided extensive studies of the learning environment (e.g., Heckhausen, 1989). They highlight learners’ rational decision-making processes. Yet, both of these theoretical concepts show a predominantly cognitive approach and, therefore, they neglect emotional aspects, which are often unconscious, but very crucial for the development of learning motivation. SDT is one of the most prominent approaches, which systematically integrate emotional aspects (Ryan and Deci, 2017; see Müller et al., 2006).

### Motivational Regulation

SDT distinguishes between intrinsic motivation and four types of extrinsic motivation. Intrinsic motivation characterizes behaviors that are enjoyable or pleasant and are thus exhibited “for their own sake” (Ryan and Deci, 2020). In education, intrinsic motivation is worth striving for, as it positively affects relevant outcomes such as vitality or achievement among students of different ages (Patrick et al., 2000; Chirkov and Ryan, 2001; Sheldon and Krieger, 2007; Taylor et al., 2014; Núñez and León, 2016; Thomas et al., 2018).

All behaviors characterized by extrinsic regulations are conducted because of external incentives (Ryan and Deci, 2017, 2020). Extrinsic motivational regulations differ, however, in terms of the strength of self-determination and associated outcomes (Ryan and Deci, 2020). They can be arranged according to an underlying continuum of self-determination, which is thought to be the origin of differential associations with outcome variables. *External regulation* is the outer end of the self-determination continuum. It characterizes behaviors stimulated by external pressures, contingent praise, and rewards (Ryan and Deci, 2020). Externally regulated behaviors are hardly self-determined and are associated with diminished vitality or personal growth (Ryan and Deci, 2000a; Thomas et al., 2018). *Introjected regulation* is slightly more self-determined. It concerns behaviors that are exhibited due to poorly internalized social norms. They are characterized by ego-involvement, such as pride or shame, where self-esteem is dependent on outcomes and others (Ryan and Deci, 2020; Bieg et al., 2020). Recent research suggests that a distinction between an approach and an avoidance component should be considered because introjected approach regulation is positively related to well-being, life satisfaction, and positive affect, whereas introjected avoidance regulation shows insignificant associations with these outcomes (Assor et al., 2009; Sheldon et al., 2017). *Identified regulation* goes along with relatively high self-determination and characterizes behaviors conducted to achieve important personal goals. However, as the values are not completely internalized, a person with identified regulation may experience inner conflict when there are other attractive behavioral alternatives (Ryan and Deci, 2000a, 2020). Nevertheless, identified regulation is a meaningful predictor of learners’ well-being, positive affect, life satisfaction, and academic achievement (Ryan and Deci, 2000a, 2020), including those at university (Sheldon et al., 2017; Thomas et al., 2018). The most self-determined type of extrinsic motivational regulation is *integrated regulation*. With integrated regulation, a behavior is conducted because of an important personal goal and without conflict because this person’s value system is mature and stable (Ryan and Deci, 2000a). Unfortunately, integrated regulation is difficult to measure with standard questionnaires (Vallerand et al., 1992) and is thus excluded from the scope of this study. Overall, intrinsic motivation and extrinsic regulations are affected by environmental conditions, specifically, how well a particular environment facilitates the satisfaction of the three basic psychological needs (Ryan and Deci, 2020).

Amotivated behaviors fall outside this framework. This type of motivational construct must be considered in order to fully understand human behavior. These “behaviours are energised and they are explicable, but they are not considered motivated because they are not regulated by intentional processes” (Deci and Ryan, 1994, p. 3). Students with high scores on amotivation perceive their behaviors as caused by forces outside their control, and start asking themselves why they attend university at all (see also Müller and Louw, 2004).

### Basic Psychological Needs Satisfaction and Frustration

SDT posits that the satisfaction of three basic psychological needs, namely autonomy, competence, and relatedness, foster intrinsic motivation and self-determined extrinsic regulations (Ryan and Deci, 2017). Autonomy is the need to experience volition regarding one’s behavior. It is satisfied when a person has free choice and room to maneuvre. As long as the desired behavior is in harmony with one’s endorsed values, he/she can experience autonomy even in situations with few or no alternatives (Ryan and Deci, 2020). Competence is the need to feel efficient and capable of bringing about change. It is satisfied when a person can rise to meet a challenge, but not when he/she completes easy tasks (Ryan and Deci, 2017). Relatedness is the need to feel connected with and cared for by other people. It can be satisfied when a person is cared for and treated with unconditional respect and appreciation (Ryan and Deci, 2000b; La Guardia and Patrick, 2008). The satisfaction of all three basic psychological needs is a predictor of vitality and autonomous types of motivation, whereas frustration predicts ill-being and controlled types of motivation (Bartholomew et al., 2011; Chen et al., 2015; Ryan and Deci, 2017). These impacts were shown empirically for university students (Cordeiro et al., 2016).

BPNS and BPNF have differential functions for psychological growth, especially in young people (Vansteenkiste and Ryan, 2013; Inguglia et al., 2018; Vansteenkiste et al., 2020). Thus, it should also be worthwhile to explore BPNS and BPNF separately for the study of motivational regulation and vitality in pandemic-induced distance learning. Learning environments can contribute toward BPNS and BPNF by supporting and/or thwarting students’ basic psychological needs. Such changes in BPNS and BPNF affect the quality of students’ motivation, well-being, and ill-being (Aelterman et al., 2019; Wang et al., 2019). BPNS can serve as a buffer in times of stress and promote psychological self-regulation, life satisfaction, and well-being (see Vansteenkiste and Ryan, 2013; Vansteenkiste et al., 2020), especially during the pandemic (e.g., Vermote et al., 2021).

### Vitality

Subjective vitality is the physical and mental energy that originates from self-direction, autonomy, and intrinsic motivation. It is a dynamic concept in health psychology and refers to individuals’ conscious experience of feeling alive and having energy (Ryan and Frederick, 1997). It represents a powerful resource that can help regulate purposive actions (Ryan and Deci, 2017). Subjective vitality is an indicator of psychological well-being (Ryan and Frederick, 1997). People who have high subjective vitality report being more active and productive, exhibiting better coping behaviors as well as experiencing more robust psychological health and well-being (Kawabata et al., 2017; Inguglia et al., 2018). Empirical evidence suggests that access to support for basic psychological needs increases subjective vitality (Vansteenkiste and Ryan, 2013). In addition, research has indicated that the relationship between BPNS or BPNF and outcomes such as vitality are mediated by motivational regulations (McDonough and Crocker, 2007; Alcaraz et al., 2015; Núñez and León, 2016). Accordingly, the present study also considered vitality and its dependence on BPNS, BPNF, and motivational regulation styles (see also Ryan et al. 2010).

## Distance Learning, Motivation, and Well-Being

The psychological impact of the pandemic can be described as wide-ranging, substantial, and probably long-lasting (Brooks et al., 2020). This also concerns learners in various educational institutions (Adnan and Anwar, 2020; Bojović et al., 2020; Li et al., 2021). During the lockdowns to contain the spread of COVID-19, students had to cope with the general situation and were confronted with a completely new virtual teaching and learning culture associated with the loss of face-to-face contact in educational settings.

Previous meta-analyses show that virtual learning environments are generally not superior to traditional teaching-learning environments in terms of cognitive, emotional, and motivational outcomes (Krammer et al., 2020). There is evidence that blended learning formats yield slightly better results in terms of student achievement (Means et al., 2013; Schneider and Preckel, 2017). Ultimately, however, the impact of virtual learning environments depends on the quality of didactic implementation and, as in traditional learning environments, on teacher actions (teacher matters, e.g., Fryer and Bovee, 2016; Arghode et al., 2017). Studies focusing on motivation in virtual learning contexts have found that virtual learning environments do not motivate *per se* (e.g., Hartnett, 2015). Empirical research has presented mixed findings with higher (Rovai et al., 2007; Réka et al., 2015) and lower (Marchand and Gutierrez, 2012) intrinsic motivation and positive emotions in distance learning (Marchand and Gutierrez, 2012). However, before the pandemic, instructors mostly offered virtual teaching voluntarily, and those who did it generally had the necessary technical know-how to run such sessions. In this respect, the findings from this period cannot be directly compared with the exceptional situation of forced distance learning during the pandemic.

Studies based on SDT and related theories show that a lack of social presence and interaction (e.g., Bowers and Kumar, 2015; Butz and Stupnisky, 2017; Wang et al., 2019) seems to be the major impairment in distance learning when compared to face-to-face learning. For example, a study comparing distance and face-to-face learning among undergraduate students (Wang et al., 2019) found that BPNS was lower and BPNF was higher in online settings than in conventional settings. Other studies have demonstrated direct (Hsu et al., 2019) and indirect (Chen and Jang, 2010) associations between BPNS and autonomous motivation in distance learning. Some studies have also suggested that the importance of relatedness in explaining autonomous forms of motivation is lower in distance learning, with autonomy and competence being strong predictors of motivation in online settings (Huang et al., 2019; Wang et al., 2019; Martinek et al., 2021). This indicates that distance learning is not *per se* associated with negative effects.

Recent studies referring to the specific situation of forced distance learning during the COVID-19 pandemic show that students generally considered virtual learning more superficial and less sustainable (Chen et al., 2021). Students reported tension, overload, worry, emotional loneliness, and reduced well-being in connection with virtual learning (Kedraka and Kaltsidis, 2020; Sahu, 2020). This was especially true for courses held in the asynchronous mode. Synchronous settings, such as interactive virtual classes, question and answer sessions, and small group discussions were considered more supportive than asynchronous ones (Gewin, 2020; Chen et al., 2021).

Based on SDT, Wong (2020) examined whether students perceived the arousal and satisfaction of their basic needs for relatedness, competence, and autonomy within an online learning context. It turned out that students’ basic psychological needs were only partially met through online learning. In particular, the need for relatedness suffered due to fewer social interactions with teachers and peers. Many students reported the lack of motivation and stress while starting their daily online sessions and had difficulty sustaining their attention (Wong, 2020). Similar to face-to-face settings, moderate associations between university students’ autonomy satisfaction and intrinsic learning motivation were found in distance learning (Holzer et al., 2021). Other studies conducted during the pandemic showed that most students voted against the notion that online learning is more motivating than conventional learning (Adnan and Anwar, 2020). They argued that face-to-face contact is beneficial for learning in general (see also Händel et al., 2020 or Kedraka and Kaltsidis, 2020; Organization for Economic Co-operation and Development: OECD, 2020).

Very few studies since the inception of the pandemic have explicitly investigated motivation, BPNS, and vitality, as well as associations among these constructs. Arslan (2021) showed that, for example, loneliness and subjective vitality of college students are negatively correlated. Individuals with certain, relatively stable characteristics like morning-orientation, conscientiousness, openness, and low neuroticism seemed to cope better with the lockdown with respect to outcomes such as vitality, self-determined motivation, and BPNS (Staller et al., 2021).

## Research Questions and Hypotheses

This paper addresses differences in university students’ motivational regulation styles, BPNS, BPNF, and vitality before and after the switch to distance learning during the lockdowns to contain the spread of COVID-19. According to the empirical findings reported above, autonomous motivation was assumed to be lower and controlled forms of motivation to be higher in forced distance learning settings during the COVID-19 pandemic. Owing to its higher autonomy and conceptual reference to self-esteem-enhancing contingencies, we assumed that introjected approach regulation would be lower during pandemic-induced distance learning than during face-to-face learning conducted previously. Somewhat higher values were expected to result from forced distance learning for introjected avoidance regulation. It was also assumed that the students experienced less BPNS and more BPNF during the distance learning phase. We expected relatedness to both peers and faculty members to be lower during forced distance learning (Wong, 2020). Research has also indicated that vitality decreased during the pandemic when compared to the period before it (e.g., Martínez-González et al., 2021).

We assumed that BPNS and BPNF can explain motivational regulation during forced distance learning. We examined the predictive power of each basic psychological need on the investigated motivational regulations separately. The structural equation model also tests the extent to which motivational regulation serves as a mediator between BPNS (and BPNF) and students’ vitality (McDonough and Crocker, 2007; Alcaraz et al., 2015). Owing to the high intercorrelations between BPNS and BPNF, we drew up separate models for each.

### Hypotheses

1. The autonomous forms of motivation (intrinsic and identified regulation) are lower during forced distance learning than during regular face-to-face learning. Moderate to high effects are expected because both intrinsic motivation and identified regulation are sensitive to environmental changes.
2. Controlled forms of motivation (introjected and external regulation) are higher during forced distance learning than they were before the change to virtual learning environments was made. However, introjected approach regulation should decline in a differentiated manner. It is assumed that differences are smaller for controlled types of motivation than for autonomous ones because the former are generally less strongly associated with environmental variables (such as BPNS or BPNF) and are therefore less affected by changes such as the abrupt switch to distance learning.
3. BPNS is lower and BPNF is higher after the switch to forced distance learning. We expect social relatedness with the peer group and faculty to be significantly lower during forced distance learning.
4. Students’ vitality is lower during forced distance learning than it was during regular face-to-face learning before the COVID-19 pandemic.
5. BPNS and BPNF explain the motivational regulations during forced distance learning. We expect correlations between BPNS and BPNF and the autonomous types of motivation to be higher than those between BPNS and BPNF and the controlled types of motivation.
6. Motivational regulations mediate the correlation between BPNS or BPNF and vitality.

## Method

### Sample

#### Sample 1 (Pre-COVID-19 Distance Learning)

Before March 2020, we collected data from 1,139 students from different study programs. Of the total sample (64% female, 34% male, and 2% no information), 45% were studying at Austrian and 55% at German universities or university colleges. The average number of completed semesters was 3.7 (*SD*=2.9). As many as 45% of the students indicated that they were pursuing a teaching degree. The others were mostly pursuing majors in the social sciences and humanities. The average age was 21.1years (*SD*=5.1). Data exist for this sample on motivational regulation (*N*=1,139), BPNS, BPNF (*N*=494), relatedness with peers and faculty (*N*=730), and subjective vitality (*N*=904).

#### Sample 2 (During Forced Distance Learning)

A sample of 1,835 students from eight universities and university colleges in Austria (56%) and Germany (44%) participated in the online survey. Data are available for all students on the constructs collected. The mean age was 23.54years (*SD*=5.77). Of the total, 79% were female, 20.9% were male, and 0.4% did not provide gender-related information. The average number of completed semesters was 5.9 (*SD*=4.8). Of the students surveyed, 56.4% were pursuing a teaching degree at the time of the survey. All other students were majoring in the social sciences and humanities. In our sample, 15.6% of the students were writing their thesis at the time of study and were therefore in one of the last semesters of their studies. Only 12.3% lived alone, 24.4% lived with one other person, 23% with two other individuals, and 23.4% with three or more. Most students (73%) were satisfied with their technical equipment for distance learning (internet, PC, etc.). Overall, the age structure and subjects studied in samples 1 and 2 were fairly similar, which justifies a comparison between both groups (cf. Martinek et al., 2021).

### Measures

To measure motivational regulation style, we used the Scales for the Measurement of Motivational Regulation for Learning in University Students (SMR-L, Thomas et al., 2018), which differentiates between two autonomous (intrinsic and identified regulations) and two controlled types (introjected and external regulation) of motivation. To measure the introjected approach and avoidance regulation separately, we added three items to the scales (cf. Bieg et al., 2020). A total of 15 items were rated on a 7-point Likert scale that ranged from *does not apply at all* (1) to *applies completely* (7). The instrument captures the following scales (item examples are given in parentheses):

– Intrinsic regulation (*I really enjoy learning in my studies*).
– Identified regulation (*I am committed to my studies in order to achieve the goals I have set for myself*).
– Introjected Regulation (“Approach”: *I want to show myself that I can be successful in my studies*; “Avoidance”: *I am currently studying because otherwise I would have a guilty conscience*).
– External regulation (*I study primarily because I cannot get a well-paid job without an academic qualification*).

The items generally refer to face-to-face learning, which is why the word “online study” was used to define the distance learning context. The internal consistencies of the scales (Cronbach’s alpha) were satisfactory (see Table 1). Confirmatory factor analyses revealed that the five-dimensionality of the instrument was superior to the two-dimensional factor solution (autonomous and controlled regulation) and the four-dimensional variant (without differentiation of introjected regulation): Five-factor solution: χ^2^(76)=390.503, *p*<0.01, *CFI*=0.975, *RMSEA*=0.035 (see also Thomas et al., 2018). The simplex structure of motivational regulation styles can be replicated (see the correlations).

**Table 1:** Motivational regulations before and during the forced distance learning period.

			Before	During				
			distance learning					
			(*N* =1,139)	(*N* =1,835)				
Scale	Items	α	M (SD)	M (SD)	t^1^	df	p	Cohen’s d
Intrinsic motivation	3	0.84/0.90	4.67 (1.22)	3.32 (1.70)	−19.00	2,940	<0.001	0.90
Identified regulation	3	0.75/0.75	5.39 (1.14)	4.83 (1.36)	−8.54	2,940	<0.001	0.39
Introjected regulation								
Approach	3	0.74/0.79	4.60 (1.44)	4.30 (1.57)	−2.11	2,931	<0.001	0.10
Avoidance	2	0.61/0.72	3.49 (1.45)	3.73 (1.67)	9.12	2,931	<0.001	0.42
External regulation	3	0.75/0.70	4.30 (1.52)	4.51 (1.47)	2.32	2,937	<0.001	0.10

Scale: 1=does not apply at all, 7=applies completely.

1 *t*-test, Scheffé procedure for independent samples.

BPNS and BPNF were assessed with the German version of the Basic Psychological Needs Satisfaction and Frustration Scale (German version: Heissel et al., 2018; original version: Chen et al., 2015). The sample reliabilities were at least satisfactory, ranging from 0.70 to 0.90 (Table 2). A total of 24 items was rated on a 5-point Likert scale ranging from *does not apply at all* (1) to *applies completely* (5). A confirmatory factor analysis (CFA) revealed good factor validity [BPNS: χ^2^(47)=217.194, *p*<0.01, *CFI*=0.975, *RMSEA*=0.032; BPNF: χ^2^(47)=217.194, *p*<0.01, *CFI*=0.980, *RMSEA*=0.032]. The instrument captures the following scales (item examples are given in parentheses):

– BPNS Autonomy (*I feel free to choose what I do in my studies*).
– BPNS Competence (*I currently feel competent in my studies*).
– BPNS Relatedness (*I feel related to the people I spend time with during my studies*).
– BPNF Autonomy (*I currently feel forced to do many things in the course of my studies that I would not choose for myself*).
– BPNF Competence (*I am currently unsure of my abilities in relation to studying)*.
– BPNF Relatedness (*I feel that the relationships I have made in the course of my studies are superficial*).

**Table 2:** Basic psychological need satisfaction (BPNS) and basic psychological needs frustration (BPNF) before and during the distance learning period.

			Before	During				
			distance learning					
			(*N* =494)	(*N* =1,177)				
Scale	Items	α	M (SD)	M (SD)	t^1^	df	p	Cohen’s d
BPNS autonomy	4	0.76/0.72	3.51 (0.76)	3.14 (0.86)	08.71	2,227	<0.001	0.45
BPNS competence	4	0.88/0.90	4.00 (0.71)	3.28 (0.98)	14.94	2,229	<0.001	0.76
BPNS relatedness	4	0.84/0.79	4.32 (0.74)	3.07 (0.94)	27.11	2,228	<0.001	1.38
BPNF autonomy	4	0.84/0.84	2.75 (1.03)	3.60 (1.00)	−16.45	2,228	<0.001	0.84
BPNF competence	4	0.79/0.84	1.56 (0.66)	2.32 (1.04)	−15.50	2,227	<0.001	0.79
BPNF relatedness	4	0.74/0.70	1.81 (0.79)	1.99 (0.81)	−04.41	2,228	<0.001	0.23

Scale: 1=does not apply at all, 7=applies completely.

1 *t*-test, Scheffé procedure for independent samples.

A well-established scale for the assessment of relatedness with peers and faculty was used (Müller and Thomas, 2018). The perceived relatedness with the peer group (e.g., I feel accepted by my fellow students) and the faculty (e.g., I have good contact with the lecturers) were measured with three and four items, respectively (Table 3). The items were answered on a 7-point Likert scale ranging from *strongly disagree* (1) to *strongly agree* (7). Internal consistency was satisfactory for both scales (α=0.79 and α=0.84, respectively). *CFA* provided a clear two-dimensional structure and was superior to a one-dimensional version [χ^2^(11)=35.308, *p*<0.01, *CFI*=0.986, *RMSEA*=0.025].

**Table 3:** Relatedness with peers and with faculty and vitality before and during the distance learning period.

			Before	During				
			distance learning					
			(*N* =730/571^2^)	(*N* =1,736)				
Scale	Items	α	*M* (*SD*)	*M* (*SD*)	*t* ^1^	*df*	*p*	Cohen’s *d*
Relatedness
Peers	3	0.79/0.80	5.61 (1.08)	3.78 (0.94)	41.33	2,422	0.00	2.11
Faculty	4	0.78/0.84	4.46 (1.24)	3.33 (0.94)	24.53	2,424	0.00	1.25
Vitality	6	0.91/0.93	3.83 (1.19)	4.18 (1.36)	−5.35	2,322	0.00	0.27

Scale: 1=strongly disagree to 7=strongly agree.

1 *t*-test, Scheffé procedure for independent samples.

2 There are 571 records for the vitality scale before distance learning.

The German version of the Subjective Vitality Scale (Ryan and Frederick, 1997) was used to measure vitality (Table 3). The participants answered prompts (e.g., I do not feel very dynamic at the moment) on a 7-point Likert-type scale from *not true* (1) to *very true* (7). The instrument achieved good levels of scale reliability (α=0.92) and factor validity [χ^2^(14)=95.730, *p*<0.01, *CFI*=0.992, *RMSEA*=0.041].

### Statistical Analyses

**Figure 1:**
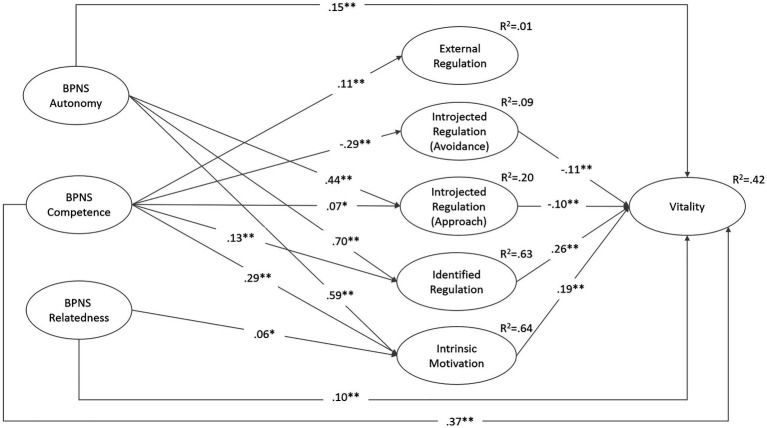
Structural equation model with BPN-Satisfaction, motivational regulation, and vitality (during the forced distance learning period). χ^2^(488)=1667.328, *p*<0.01, *CFI*=0.965, *RMSEA*=0.026. Variables are modelled latently; measurement models are not shown; ^*^p<0.05, ^**^p<0.01; non-significant paths are not shown. A model in which social relatedness was differentiated according to peers and faculty did not yield any additional explanatory value for motivational regulation and vitality. In addition, the fit indices were slightly worse [χ^2^(360)=1440.024, *p*<0.01, *CFI*=0.959, *RMSEA*=0.029].

**Figure 2:**
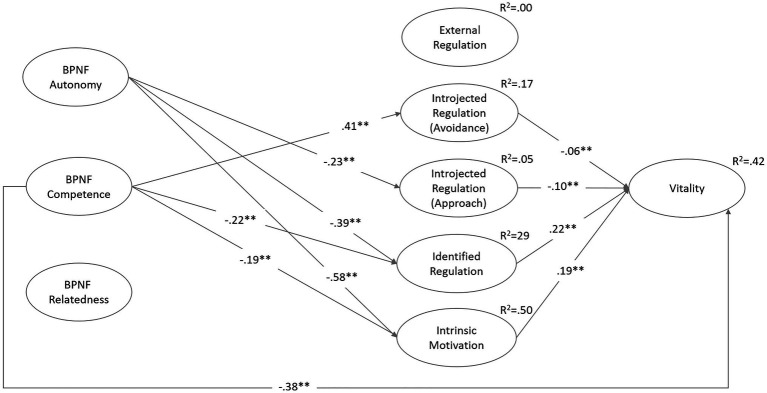
Structural equation model with BPN-Frustration, motivational regulation and vitality (during the forced distance learning period). χ^2^(494)=1626.855, *p*<0.01, *CFI*=0.965, *RMSEA*=0.027. Variables are modelled latently; measurement models are not shown; ^*^p<0.05, ^**^p<0.01; non-significant paths are not shown.

To test possible mean differences described in hypotheses 1 to 4, we used a t-test (procedure Scheffé). To analyze linear associations, we used Pearson correlations (Table 4). To test hypotheses 5 and 6, we computed two structural equation models using Amos 25.0 software (cf. Byrne, 2016) and standard fit indices (Figures 1, 2). Model goodness of fit was checked using root-mean-square error of approximation (*RMSEA*) and comparative fit index (*CFI*). Though the χ^2^ statistic was reported, it had the disadvantage of being sensitive to sample size. For *RMSEA*, values above 0.05 and below 0.08 are considered good and acceptable, respectively, and for the *CFI*, values above 0.95 but at least 0.90 should be achieved (Kline, 2011).

**Table 4:** Pearson correlations between all relevant variables (during distance learning).

	1	2	3	4	5	6	7	8	9	10	11	12	13
1. Intrinsic motivation	—												
2. Identified Regulation	0.57^**^	—											
3. Introjected Regulation (Approach)	0.34^**^	0.50^**^	—										
4. Introjected Regulation (Avoidance)	−0.18^**^	−0.04	0.32^**^	—									
5. External Regulation	0.06^*^	0.06^*^	0.27^**^	0.25^**^	—								
6. BPNS Autonomy	0.61^**^	−0.06^*^	0.30^**^	−0.18^**^	−0.01	—							
7. BPNS Competence	0.59^**^	0.45^**^	0.22^**^	−0.25^**^	0.03	0.57^**^	—						
8. BPNS Relatedness	0.23^**^	0.37^**^	0.13^**^	−0.09^**^	−0.03	0.32^**^	0.35^**^	—					
9. BPNS Relatedness (faculty)	0.45 ^**^	0.10^**^	0.17^**^	−0.07^**^	−0.02	0.57^**^	0.50^**^	0.28^**^	—				
10. BPNS Relatedness (peers)	0.17^**^	−0.28^**^	0.11^**^	−0.08^**^	−0.03	0.25^**^	0.33^**^	0.65^**^	0.32^**^	—			
11. BPNF Autonomy	−0.56 ^**^	−0.39^**^	−0.14^**^	0.25^**^	0.07^**^	−0.63^**^	−0.51^**^	−0.18^**^	−0.43^**^	−0.14^**^	—		
12. BPNF Competence	−0.45^**^	−0.35^**^	−0.09^**^	0.33^**^	0.04	−0.45^**^	−0.79^**^	−0.30^**^	−0.40^**^	−0.29^**^	0.46^**^	—	
13. BSNF Relatedness	−0.17^**^	−0.15^**^	−0.01	0.19^**^	0.10^**^	−0.25^**^	−0.43^**^	−0.58^**^	−0.25^**^	−0.59^**^	0.24^**^	0.44^**^	—
14. Vitality	0.48^**^	0.40^**^	−0.14^**^	−0.26^**^	−0.03	0.42	0.55^**^	0.29^**^	0.32^**^	0.26^**^	−0.42^**^	−0.51^**^	−0.30^**^

* *p* <0.05;

** *p* <0.01.

### Procedure

Following the switch to forced distance learning and teaching during the COVID-19 pandemic, the students were surveyed using an online questionnaire between April and June 2020. Students were reached *via* individual online lectures. The completion of the online questionnaires took about 15 to 20min on average. Before participating in the survey, the students were assured anonymity. They were given the option of retracting study participation or withdrawing at any time without any penalty. The students gave their consent to the use of their data for research purposes.

## Results

The mean values of all five motivational regulation types differed significantly before and after the introduction of forced distance learning (Table 1). In line with hypothesis 1, the students showed lower intrinsic motivation and identified regulation during the forced distance learning period. They also reported higher levels of controlled motivation and introjected approach regulation during forced distance learning.

BPNS and BPNF showed a significant difference in mean values in a comparison of the surveys conducted before and during the COVID-19 pandemic (see Table 2). The satisfaction of the basic needs for competence and relatedness were substantially lower during the forced distance learning period (*d*=0.76 and 1.38, respectively). A different picture emerged for the BPNF when compared with the BPNS. The needs for autonomy and competence were obviously more frustrated during forced distance learning than before. According to the effect size, the frustration of relatedness differed only slightly.

A differentiated view of relatedness presents a distinction between relatedness with peers and faculty (Table 3). In both cases, there is a significant difference in terms of the disadvantage of forced distance learning. This difference is especially large for relatedness with peers. Relatedness with faculty was significantly lower during forced distance learning than before the changeover.

It was also assumed that the vitality of the students would be lower during forced distance learning than before. The results show that subjective vitality was somewhat higher during this time, although the effect size was small. This was, however, not true for first-semester students, for whom vitality during forced distance learning turned out to be lower than for students in other semesters [first semester: M=3.58, SD=1.14; two or more semesters: M=4.20, SD=1.37; t(1811)=−3.743, *p*<0.01].

The following structural equation models were calculated to test hypotheses 5 and 6. In Figure 1, we modelled BPNS as a predictor of motivational regulatory style and vitality for all students in sample 2 (during the forced distance learning period). To avoid multicollinearity, relatedness was modelled as one variable and not differentiated into relatedness with peers and faculty. The differentiated modelling of relatedness did not provide any added explanatory value in the model.

The results show that variance in intrinsic motivation and identified regulation was well explained by BPNS (*R*^2^=0.64 and 0.63, respectively). Autonomy (β=0.59 and 0.70, both *p*<0.01) and, to a lesser extent, competence (β=0.29 and 0.13, both *p*<0.01) both qualified as predictors of autonomous types of motivational regulation. Relatedness showed a small association with intrinsic motivation. The variance in the introjected approach regulation could be explained to 20%, with autonomy having the highest proportion (β=0.44, *p*<0.01). Introjected avoidance regulation was negatively associated with the satisfaction of the need for competence; however, external regulations could hardly be explained in this model (see Figure 1). Overall, 42% of the variance of the students’ vitality was explained in this model, with the autonomous forms of motivation and the two scales of introjected regulation being positively and negatively associated with vitality, respectively. The satisfaction of all three basic needs directly explained vitality, with competence showing the highest explanatory value (β=0.37, *p*<0.01).

Figure 2 presents the relationship among the investigated variables with each BPNF as independent variables for the explanation of motivational regulation during forced distance learning. Consistent with our expectations in hypothesis 5, the autonomous types of motivational and introjected approach regulation were negatively associated with the frustration of autonomy. The more the need for competence was frustrated, the lesser the students reported identified regulation (β=−0.22, *p*<0.01) and intrinsic motivation (β=−0.19, *p*<0.01). The learners’ introjected avoidance regulation was explained by the frustration of competence (β=0.41, *p*<0.01) and thus better than by the BPNS. The frustration of relatedness could not explain any motivational regulation or vitality. As in in Model 1, external regulation was not explained in this model.

## Discussion

Based on SDT, this study investigated the extent to which university students’ BPNS, BPNF, motivational regulation, and vitality differed between regular on-site education before COVID-19 and the initial phase of forced distance learning during the COVID-19 lockdown. We argued that the short-term and forced changeover to distance learning led to changes in basic support for psychological needs, thus curtailing BPNS and triggering BPNF. These changes were associated with low autonomous motivation. In line with our expectations as articulated in hypothesis 1, the mean scores of intrinsic motivation and identified regulation were considerably lower during forced distance learning than they were before (cf. Wong, 2020). The results show that the differentiation of introjected regulation was worthwhile, as approach and avoidance aspects were associated with distinct findings (see also Gagné et al., 2014). Introjected approach regulation, which is higher in self-determination than introjected avoidance regulation (see Table 2), was slightly lower during the forced distance learning period than during regular on-site learning, whereas introjected avoidance regulation was significantly higher during this time (confirmation of hypothesis 2). These findings suggest that students’ motivational regulation during the pandemic was characterized by the avoidance of negative feelings such as shame or a guilty conscience. In line with other studies that used a differentiated assessment of introjected regulation, our results show that this differentiation is adequate and necessary (cf. Assor et al., 2009; Howard et al., 2017; Sheldon et al., 2017; Bieg et al., 2020). If introjected regulation is conceptualized as a single measure, it can yield indifferent, varying, and therefore difficult-to-interpret associations with outcome variables (e.g., Ng et al., 2012). Finally, external regulation was only slightly higher during than before the change to forced distance learning (hypothesis 2, see also Rovai et al., 2007). Small associations with various antecedents suggest that when it comes to university students, this regulation style is hardly dependent on factors related to the study environment (cf. Müller and Palekčić, 2005; Thomas et al., 2018).

Consistent with our expectations in hypothesis 3, BPNS was lower and BPNF was correspondingly higher during the initial forced distance learning phase. The difference in relatedness was particularly high (Wong, 2020). Relatedness with peers was significantly lower during the forced distance learning phase (*d*=2.11). The results also show that in the field of education, a distinction between relatedness with peers and faculty is needed, as the mean scores of the two scales differed significantly (see also Müller and Thomas, 2018). The study shows only a small difference in relatedness frustration (*d*=0.21) between the pre-pandemic and forced distance learning periods. As contact was restricted during the pandemic, relatedness was not actively frustrated by both the peers and teachers in the first semester of forced distance learning. On the other hand, virtual online communications and lockdown restrictions may have severely limited the satisfaction of social contacts and thus led to attenuated social relatedness. This may explain why the satisfaction of relatedness was considerably lower during forced distance learning than during regular face-to-face learning period, whereas the frustration of this need was only marginally higher.

The slightly higher subjective vitality of the students during the forced distance learning period was perhaps the most surprising finding. Thus, hypothesis 4 could not be confirmed. However, a detailed look at the data shows that vitality was significantly lower for the first-semester students during forced distance learning period than for students in higher semesters. This was probably due to the fact that these students were confronted with two challenges: the beginning of a new stage of life and the restrictions because of the pandemic. This is also reflected, for example, in the significantly lower scores of the first-semester students’ intrinsic motivation and the satisfaction of their needs for competence and relatedness. Starting their studies under these conditions appears to be suboptimal from the students’ perspective. This emphasizes the importance of providing special support for this group of students as well as the need to further investigate the motivational development of students who began their studies during the pandemic.

We also investigated whether vitality during the forced distance learning period can be explained by BPNS and BPNF as well as the extent to which the regulatory motivational types are appropriate mediators in this relationship (hypotheses 5 and 6; see also Núñez and León, 2016 or Martinek et al., 2021). In line with our expectations, the results show that the satisfaction of autonomy and competence are strong predictors of self-determined types of motivation (cf. e.g. Hsu et al., 2019; Vansteenkiste et al., 2020). Associations for introjected approach regulation were similar to those of other autonomous motivational regulations, being positively associated with the satisfaction of autonomy and competence and negatively associated with autonomy frustration. As a less self-determined form of extrinsic motivation, introjected avoidance regulation was negatively correlated with competence satisfaction and is explained by competence frustration. These findings correspond with results from studies conducted before the pandemic that showed associations between BPNS and introjected approach regulation and between BPNF and introjected avoidance regulation (Gagné et al., 2014; Cheon et al., 2019). The finding that satisfaction of relatedness only marginally predicted motivational regulation in the forced distance learning setting during COVID-19 was somewhat surprising. Even differentiating between relatedness with students and faculty did not change this small effect. An explanation for this unexpected result may be that satisfaction of the three basic needs was intercorrelated (see Table 4). Thus, the structural equation model could also underestimate the relevance of the support of relatedness because there are significant moderate correlations between BPNS for relatedness and autonomous types of regulation (*r*=0.23 and 0.37, both *p*<0.01; see also Table 4 for the correlations between all basic needs). Our results corroborate the findings of Holzer et al. (2021), who also found little or no effects of relatedness on intrinsic motivation among university students during the forced distance learning period. They argued that social withdrawal in the sense of “cocooning” did not necessarily have a negative effect on internal psychological regulations and well-being. Online situations with no direct social interactions may well be experienced as highly self-determined (e.g., Ryan et al., 2006, 2010). If the other two basic psychological needs for autonomy and competence are reasonably satisfied, students may still be “well aligned with their inner selves” (Martinek, et al., 2021) and the need for relatedness may play a subordinate role. Another reason could be that the basic need for relatedness is not predominantly satisfied in the study context, but by family and/or friends (Legault et al., 2006; Tezci et al., 2017). Accordingly, the social connection to fellow students or the teaching staff would be less important for one’s internal psychological regulation and well-being. These explanations are tentative and future research should examine the role of relatedness for motivational regulation and well-being over longer periods of distance learning.

Our study shows that BPNS and BPNF are hardly able to explain external regulations. The phenomenon that autonomous forms of motivation are better explained by perceived needs satisfaction or frustration is well known (e.g., Vandenkerckhove et al., 2019; Kaiser et al., 2020). In both structural equation models, BPNS and BPNF explain 42% of vitality, either directly or indirectly (mediated by motivational regulations). As assumed in hypothesis 6 and in line with previous findings (McDonoug and Crocker, 2007), autonomous forms of motivation (intrinsic and identified) positively predict vitality in both models. Competence satisfaction and frustration contribute substantially to student vitality. This is consistent with other findings in the context of higher education (e.g., Levesque et al., 2004.). Although introjected approach regulation is positively related to autonomy satisfaction and negatively related to frustration, thus suggesting a relatively high level of self-determination, its association with vitality is a negative one. From our perspective, this finding highlights the position of introjected approach regulation at the edge of autonomous versus controlled motivation. In contrast, introjected avoidance regulation is a controlled type of motivation as it is negatively associated with both needs satisfaction and subjective vitality (i.e., with predictors and outcomes; see also Sheldon et al., 2017). However, study-related BPNS, BPNF, and motivational regulations are not the only predictors of general vitality. For example, individual- and/or lifestyle-related variables such as individual time management or greater amount of free time can serve as additional predictors of vitality and should be considered in future research.

Our findings emphasize the importance of high BPNS and low BPNF in distance learning settings. As is the case during forced distance learning, the support of basic psychological needs and the avoidance of needs frustration may be particularly important in other situations involving a high degree of strain.

### Limitations and Future Research

One limitation of this study is that the data are not longitudinal. As the two samples (students before and after the introduction of forced distance learning) are similar in terms of age, gender, and courses of study, the results are nevertheless meaningful. However, as the sample comprises mainly students of teacher education, the social sciences, and humanities, it is unclear whether the findings can be applied to other courses of study. The sample is largely self-selective, which affects its representativeness. As most of the data were collected in the context of (partly compulsory) courses in which most students competed surveys, the self-selection effects are probably small.

The data for sample 2 were collected in spring and summer of 2020. It is quite possible that a certain habituation effect had set in among the students or that coping strategies had been developed so that autonomous motivation or the perception of needs satisfaction became high again. On the other hand, because of the long duration of the restrictions, the students and teachers’ needs may have been even less satisfied and their motivation may have therefore suffered even more as the distance learning period progressed (cf. e.g. Marx et al., 2021). Further research must be conducted to clarify this aspect, ideally using a longitudinal design. Research can also consider relatively stable personality traits, motivational orientations, personality, and self-control abilities, which—in terms of resilience—may explain motivation and outcomes such as vitality (e.g., Vansteenkiste and Ryan, 2013; Vansteenkiste et al., 2020). It can also be worthwhile to survey amotivation, as this motivational type can be higher in times of crisis and offer valuable insights into university dropouts. Finally, one of the bigger challenges that is not just specific to the distance education context in times of a pandemic seems to be the targeting of social relatedness. Here, new approaches must be tested and, if necessary, the limits of virtual learning environments with respect to the quality of social interaction must be accepted.

### Educational Implications

Our results show that the satisfaction of basic psychological needs was significantly lower and the frustration thereof substantially higher during the forced distance learning phase than before the pandemic. This suggests that students’ basic needs should to be taken into account in the design of digital learning environments. Various measures can be taken by lecturers for this purpose. Regarding the basic need for autonomy, choices and rationales that emphasize the relevance of topics or actions in the seminar or lecture can be provided (Reeve, 2015). With regard to the need for competence, tasks with varying levels of challenge might be offered so that each student can reach a balance between his/her abilities and a given task (see Reeve, 2015; Ryan & Deci, 2017). With respect to the need for relatedness, lecturers should facilitate social interactions and collaborative work processes during their seminars. If such measures are taken and the students perceive their needs to be satisfied as a result, the highly pronounced externally determined motivation and the low level of self-determined motivation that became apparent during the pandemic can be counteracted at the same time.

### Conclusion

This study found that autonomous forms of motivation and BPNS were lower and controlled forms of motivation and BPNF were higher during the forced distance learning phase brought on by the worldwide COVID-19 pandemic than in regular face-to-face learning situations. As outlined above, it is important to support the satisfaction of students’ autonomy and competence both within and beyond distance learning settings, as they directly affect learners’ quality of motivation and are responsible for their well-being. The satisfaction of relatedness with peers and faculty was very low during the pandemic. Although it was not strongly associated with autonomous types of motivational regulation, there may be detrimental effects if the restrictions on social contact are long lasting.

## Data Availability Statement

The raw data supporting the conclusions of this article will be made available by the authors, without undue reservation.

## Ethics Statement

In Austria and Germany, it is not necessary for members of these institutions to obtain approval from the Ethics Committee for such surveys of adult persons at higher education institutions. The participants provided their written informed consent to participate in this study.

## Author Contributions

FM, AT, MC, AE, NG, DM, MW, A-KD, and SB conceptualized and designed the study and agreed on the final submission of the study. All authors performed data collection. FM carried out the statistical calculations. FM, AT, and SB wrote the first draft of the manuscript. FM managed and oversaw the entire project. All authors contributed to the article and approved the submitted version.

## Funding

This project is part of the “Qualitätsoffensive Lehrerbildung,” a joint initiative of the Federal Government and the *Länder* which aims to improve the quality of teacher training. The program is funded by the Federal Ministry of Education and Research (funding code: 01JA1908). The authors are responsible for the content of this publication.

## Conflict of Interest

The authors declare that the research was conducted in the absence of any commercial and financial relationships that can be construed as potential conflicts of interest.

## Publisher’s Note

All claims expressed in this article are solely those of the authors and do not necessarily represent those of their affiliated organizations, or those of the publisher, the editors and the reviewers. Any product that may be evaluated in this article, or claim that may be made by its manufacturer, is not guaranteed or endorsed by the publisher.

## References

[ref1] Adnan M. Anwar K. (2020). Online learning amid the COVID-19 pandemic. Students’ perspectives. J. Pedagog. Soc. Psychol. 2, 45–51. doi: 10.33902/JPSP.2020261309

[ref2] Aelterman N. Vansteenkiste M. Haerens L. Soenens B. Fontaine J. R. J. Reeve J. (2019). Toward an integrative and fine-grained insight in motivating and demotivating teaching styles. The merits of a circumplex approach. J. Educ. Psychol. 111, 497–521. doi: 10.1037/edu0000293

[ref3] Alcaraz S. Torregrosa M. Viladrich C. (2015). How coaches’ motivations mediate Between basic psychological needs and well-being/ill-being. Res. Quart. Exer. Sport 86, 292–302. doi: 10.1080/02701367.2015.1049691 26230963

[ref501] Arghode V. Brieger E. W. McLean G. N. (2017). Adult learning theories: Implications for online instruction. Eur. J. Train. Dev. 41, 593–609. doi: 10.1108/EJTD-02-2017-0014

[ref4] Arslan G. (2021). Loneliness, college belongingness, subjective vitality, and psychological adjustment during coronavirus pandemic. Development of the college belongingness questionnaire. J. Pos. School Psychol. 5, 17–31. doi: 10.47602/jpsp.v5i1.240

[ref5] Assor A. Vansteenkiste M. Kaplan A. (2009). Identified versus introjected approach and introjected avoidance motivations in school and in sports. The limited benefits of self-worth strivings. J. Educ. Psychol. 101, 482–497. doi: 10.1037/a0014236

[ref6] Bartholomew K. J. Ntoumanis N. Ryan R. M. Bosch J. A. Thøgersen-Ntoumani C. (2011). Self-determination theory and diminished functioning. Personal. Soc. Psychol. Bull. 37, 1459–1473. doi: 10.1177/0146167211413125, PMID: 21700794

[ref7] Baumeister R. F. (2016). Toward a general theory of motivation: problems, challenges, opportunities, and the big picture. Motiv. Emot. 40, 1–10. doi: 10.1007/s11031-015-9521-y

[ref8] Bieg S. Thomas A. Müller F. H. (2020). “A differentiated look at introjected regulation.” in Tenth SELF international Conference. 2020; Quebec, Canada.

[ref9] Bojović Ž. Bojović P. D. Vujošević D. Šuh J. (2020). Education in times of crisis. Rapid transition to distance learning. Comput. Appl. Eng. Educ. 28, 1467–1489. doi: 10.1002/cae.22318 PMC7461294 38607824

[ref10] Bowers J. Kumar P. (2015). Students' perceptions of teaching and social presence. Int. J. Web-Based Learn. Teach. Techn. 10, 27–44. doi: 10.4018/ijwltt.2015010103

[ref11] Brooks S. K. Webster R. K. Smith L. E. Woodland L. Wessely S. Greenberg N.. (2020). The psychological impact of quarantine and how to reduce it. Rapid review of the evidence. Lancet 395, 912–920. doi: 10.1016/S0140-6736(20)30460-8, PMID: 32112714 PMC7158942

[ref12] Butz N. T. Stupnisky R. H. (2017). Improving student relatedness through an online discussion intervention. The application of self-determination theory in synchronous hybrid programs. Comp. Educ. 114, 117–138. doi: 10.1016/j.compedu.2017.06.006

[ref13] Byrne B. M. (2016). Structural Equation Modeling With AMOS: Routledge.

[ref14] Chen K.-C. Jang S.-J. (2010). Motivation in online learning. Testing a model of self-determination theory. Comp. Human Behav. 26, 741–752. doi: 10.1016/j.chb.2010.01.011

[ref15] Chen E. Kaczmarek K. Ohyama H. (2021). Student perceptions of distance learning strategies during COVID-19. J. Dent. Educ. 85, 1190–1191. doi: 10.1002/jdd.12339, PMID: 32686154 PMC7404836

[ref16] Chen B. Vansteenkiste M. Beyers W. Boone L. Deci E. L. van der Kaap-Deeder J.. (2015). Basic psychological need satisfaction, need frustration, and need strength across four cultures. Motiv. Emot. 39, 216–236. doi: 10.1007/s11031-014-9450-1

[ref17] Cheon S. H. Reeve J. Lee Y. Ntoumanis N. Gillet N. Kim B. R.. (2019). Expanding autonomy psychological need states from two (satisfaction, frustration) to three (dissatisfaction). A classroom-based intervention study. J. Educ. Psychol. 111, 685–702. doi: 10.1037/edu0000306

[ref18] Chirkov V. I. Ryan R. M. (2001). Parent and teacher autonomy-support in Russian and U.S. adolescents. J. Cross-Cult. Psychol. 32, 618–635. doi: 10.1177/0022022101032005006

[ref19] Chiu T. K. F. (2021). Applying the self-determination theory (SDT) to explain student engagement in online learning during the COVID-19 pandemic. J. Res. Technol. Educ. 36, 1–17. doi: 10.1080/15391523.2021.1891998

[ref20] Cordeiro P. Paixão P. Lens W. Lacante M. Luyckx K. (2016). The Portuguese validation of the basic psychological need satisfaction and frustration scale. Concurrent and longitudinal relations to well-being and ill-being. Psychol. Belg. 56, 193–209. doi: 10.5334/pb.252, PMID: 30479436 PMC5853851

[ref502] Deci E. L. Ryan R. M. (1994). Promoting self-determinated education. Scand. J. Educ. Res. 38, 3–14.

[ref21] Fryer L. K. Bovee H. N. (2016). Supporting students' motivation for e-learning. Teachers matter on and off line. Internet High. Educ. 30, 21–29. doi: 10.1016/j.iheduc.2016.03.003

[ref22] Gagné M. Forest J. Vansteenkiste M. Crevier-Braud L. van den Broeck A. Aspeli A. K.. (2014). The multidimensional work motivation scale. Validation evidence in seven languages and nine countries. Eur. J. Work Org. Psychol. 24, 178–196. doi: 10.1080/1359432X.2013.877892

[ref23] Gewin V. (2020). Five tips for moving teaching online as COVID-19 takes hold. Nature 580, 295–296. doi: 10.1038/d41586-020-00896-7, PMID: 32210377

[ref24] Händel M. Stephan M. Gläser-Zikuda M. Kopp B. Bedenlier S. Ziegler A. (2020). Digital readiness and its effects on higher education students’ socio-emotional perceptions in the context of the COVID-19 pandemic. J. Res. Technol. Educ. 17, 1–13. doi: 10.1080/15391523.2020.1846147

[ref25] Hartnett M. K. (2015). Influences that undermine learners’ perceptions of autonomy, competence and relatedness in an online context. Aus. J. Educ. Technol. 31, 86–99. doi: 10.14742/ajet.1526

[ref26] Heckhausen H. (1989). Motivation Und Handeln [Motivation and Action]. Berlin: Springer.

[ref27] Heissel A. Pietrek A. Flunger B. Fydrich T. Rapp M. A. Heinzel S.. (2018). The validation of the German basic psychological need satisfaction and frustration scale in the context of mental health. Eur. J. Health Psychol. 25, 119–132. doi: 10.1027/2512-8442/a000017

[ref28] Holzer J. Lüftenegger M. Korlat S. Pelikan E. Salmela-Aro K. Spiel C.. (2021). Higher education in times of COVID-19. University students’ basic need satisfaction, self-regulated learning, and well-being. AERA Open 7:23328584211003164. doi: 10.1177/23328584211003164, PMID: 34192126 PMC7961159

[ref29] Howard J. L. Gagné M. Bureau J. S. (2017). Testing a continuum structure of self-determined motivation. A meta-analysis. Psychol. Bull. 143, 1346–1377. doi: 10.1037/bul0000125, PMID: 29048175

[ref30] Hsu H.-C. K. Wang C. V. Levesque-Bristol C. (2019). Reexamining the impact of self-determination theory on learning outcomes in the online learning environment. Educ. Inf. Technol. 24, 2159–2174. doi: 10.1007/s10639-019-09863-w

[ref31] Huang Y.-C. Backman S. J. Backman K. F. McGuire F. A. Moore D. (2019). An investigation of motivation and experience in virtual learning environments. A self-determination theory. Educ. Inf. Technol. 24, 591–611. doi: 10.1007/s10639-018-9784-5

[ref32] Inguglia C. Liga F. Lo Coco A. Musso P. Ingoglia S. (2018). Satisfaction and frustration of autonomy and relatedness needs: associations with parenting dimensions and psychological functioning. Motiv. Emot. 42, 691–705. doi: 10.1007/s11031-018-9702-6

[ref33] Kaiser L.-M. Großmann N. Wilde M. (2020). The relationship between students’ motivation and their perceived amount of basic psychological need satisfaction – a differentiated investigation of students’ quality of motivation regarding biology. Int. J. Sci. Educ. 42, 2801–2818. doi: 10.1080/09500693.2020.1836690

[ref503] Kaparounaki C. K. Patsali M. E. Mousa D. V. Papadopoulou E. V. K. Papadopoulou K. K. K. Fountoulakis K. N.. (2020). University students’ mental health amidst the COVID-19 quarantine in Greece. Psychiatry Res. 290, 113–111. doi: 10.1016/j.psychres.2020.113111, PMID: 32450416 PMC7236729

[ref34] Kawabata M. Yamazaki F. Guo D. W. Chatzisarantis N. L. D. (2017). Advancement of the subjective vitality scale. Examination of alternative measurement models for Japanese and Singaporeans. Scand. J. Med. Sci. Sports 27, 1793–1800. doi: 10.1111/sms.12760, PMID: 27704634

[ref35] Kedraka K. Kaltsidis C. (2020). Effects of the COVID-19 pandemic on university pedagogy. Students experiences and considerations. Eur. J. Environ. Sci. 7. doi: 10.46827/ejes.v7i8.3176

[ref36] Kline R. B. (2011). Principles and Practice of Structural Equation Modeling. New York: Guilford Press.

[ref37] Krammer G. Pflanzl B. Matischek-Jauk M. (2020). Aspekte der online-Lehre und deren Zusammenhang mit positivem Erleben und motivation bei Lehramtsstudierenden. Mixed-method Befunde zu Beginn von COVID-19 [aspects of online teaching and their connection with positive experience and motivation among student teachers]. Z. f. Bildungsforsch. 10, 337–375. doi: 10.1007/s35834-020-00283-2 PMC7756194 38624601

[ref38] La Guardia J. G. Patrick H. (2008). Self-determination theory as a fundamental theory of close relationships. Can. Psychol. 49, 201–209. doi: 10.1037/a0012760

[ref39] Legault L. Green-Demers I. Pelletier L. (2006). Why do high school students lack motivation in the classroom? Toward an understanding of academic amotivation and the role of social support. J. Educ. Psychol. 98, 567–582. doi: 10.1037/0022-0663.98.3.567

[ref40] Levesque C. Zuehlke A. N. Stanek L. R. Ryan R. M. (2004). Autonomy and competence in German and American university students. A comparative study based on self-determination theory. J. Educ. Psychol. 96, 68–84. doi: 10.1037/0022-0663.96.1.68

[ref41] Li Y. Wang Y. Jiang J. Valdimarsdóttir U. A. Fall K. Fang F.. (2021). Psychological distress among health professional students during the COVID-19 outbreak. Psychol. Med. 51, 1952–1954. doi: 10.1017/S0033291720001555, PMID: 32389148 PMC7225209

[ref42] Marchand G. C. Gutierrez A. P. (2012). The role of emotion in the learning process. Comparisons between online and face-to-face learning settings. Internet High. Educ. 15, 150–160. doi: 10.1016/j.iheduc.2011.10.001

[ref44] Martinek D. Carmignola M. Müller F. H. Bieg S. Thomas A. Eckes A.. (2021). How can students feel more vital amidst severe restrictions? Psychological needs satisfaction, motivational regulation and vitality of students during the coronavirus pandemic restrictions. Eur. J. Inv. Health Psychol. Educ. 11, 405–422. doi: 10.3390/ejihpe11020030, PMID: 34708827 PMC8314371

[ref45] Martínez-González N. Atienza F. L. Tomás I. Duda J. L. Balaguer I. (2021). The impact of coronavirus disease 2019 lockdown on athletes’ subjective vitality: The protective role of resilience and autonomous goal motives. Front. Psychol. 11:612825. doi: 10.3389/fpsyg.2020.612825, PMID: 33643114 PMC7902777

[ref46] Marx A. K. G. Schwab C. Frenzel A. C. (2021). "I'm tired of black boxes". Emotional experiences when teaching digitally in a time of pandemic. European Association for Research on Learning and Instruction (EARLI). [online conference]. European Association for Research on Learning and Instruction (EARLI).

[ref47] McDonough M. H. Crocker P. R. (2007). Testing self-determined motivation as a mediator of the relationship between psychological needs and affective and behavioral outcomes. J. Sport Exer. Psychol. 29, 645–663. doi: 10.1123/jsep.29.5.645 18089897

[ref48] Means B. Toyama Y. Murphy R. Baki M. (2013). The effectiveness of online and blended learning. A meta-analysis of the empirical literature. Teach. Coll. Rec. 115, 1–47.

[ref49] Müller F. H. Louw J. (2004). Learning environment, motivation and interest. Perspectives on self-determination theory. S. Afr. J. Psychol. 34, 169–190. doi: 10.1177/008124630403400201

[ref50] Müller F. H. Palekčić M. (2005). Bedingungen und Auswirkungen selbstbestimmt motivierten Lernens bei kroatischen Hochschulstudenten [Conditions and effects of self-determined motivated learning among Croatian university student]. Empirische Pädagogik. 134–165.

[ref51] Müller F. H. Palekčić M. Beck M. Wanninger S. (2006). Personality, motives and learning environment as predictors of self-determined learning motivation. Rev. Psychol. 12, 75–86.

[ref52] Müller F. H. Thomas A. E. (2018). “Die Bedeutung der sozialen Einbindung für die autonome motivation und das Wohlbefinden im Lehramtsstudium [The importance of social inclusion for autonomous motivation and well-being in teacher education]” in Motivierte Lehrperson werden und bleiben. Analysen aus der Perspektive der Theorie der Persönlichkeits-System-Interaktionen und der Selbstbestimmungstheorie. eds. Hofmann M. D. Müller F. H. (Münster: Waxmann), 101–122.

[ref53] Ng J. Y. Y. Ntoumanis N. Thøgersen-Ntoumani C. Deci E. L. Ryan R. M. Duda J. L.. (2012). Self-determination theory applied to health contexts. Perspect. Psychol. Sci. 7, 325–340. doi: 10.1177/1745691612447309, PMID: 26168470

[ref54] Núñez J. L. León J. (2016). The mediating effect of intrinsic motivation to learn on the relationship between student’s autonomy support and vitality and deep learning. Span. J. Psychol. 19:449. doi: 10.1017/sjp.2016.43, PMID: 27425178

[ref55] Organization of Economic, Co-operation and Development (OECD) (2020). Education responses to covid-19: Embarking digital learning and online collaboration. (Accessed May 5, 2020).

[ref56] Patrick B. C. Hisley J. Kempler T. (2000). “What's everybody so excited about?” The effects of teacher enthusiasm on student intrinsic motivation and vitality. J. Exp. Educ. 68, 217–236. doi: 10.1080/00220970009600093

[ref57] Reeve J. (2015). Understanding Motivation and Emotion (6th ed.). Hoboken, NJ: Wiley.

[ref58] Reeve J. (2016). A grand theory of motivation: why not? Motiv. Emot. 40, 31–35. doi: 10.1007/s11031-015-9538-2

[ref59] Réka J. Kármen D. Susana F. Kinga K. J. Edit M. Kinga S. (2015). Implications of motivational factors regarding the academic success of full-time and distance learning undergraduate students. A self-determination theory perspective. Procedia Soc. Behav. Sci. 187, 50–55. doi: 10.1016/j.sbspro.2015.03.010

[ref60] Richardson J. C. Koehler A. A. Besser E. D. Caskurlu S. Lim J. Mueller C. M. (2015). Conceptualizing and investigating instructor presence in online learning environments. Int. Rev. Res. Open Dist. Learn. 16, 256–297. doi: 10.19173/irrodl.v16i3.2123

[ref61] Rovai A. Ponton M. Wighting M. Baker J. (2007). A Comparative analysis of student motivation in traditional classroom and E-learning courses. Int. J. E-Learn. 6.

[ref62] Ryan R. M. Bernstein J. H. Brown K. W. (2010). Weekends, work, and well-being: psychological need satisfactions and day of the week effects on mood, vitality, and physical symptoms. J. Soc. Clin. Psychol. 29, 95–122. doi: 10.1521/jscp.2010.29.1.95

[ref63] Ryan R. M. Deci E. L. (2000a). Intrinsic and extrinsic motivations. Classic definitions and new directions. Contemp. Educ. Psychol. 25, 54–67. doi: 10.1006/ceps.1999.1020 10620381

[ref64] Ryan R. M. Deci E. L. (2000b). Basic psychological needs as a unifying concept. Psychol. Inq. 11, 319–338. doi: 10.1207/S15327965PLI1104_03

[ref65] Ryan R. M. Deci E. L. (2017). Self-Determination Theory: Basic Psychological Needs in Motivation, Development, and Wellness: New York: Guilford Press.

[ref66] Ryan R. M. Deci E. L. (2020). Intrinsic and extrinsic motivation from a self-determination theory perspective. Definitions, theory, practices, and future directions. Contemp. Educ. Psychol. 61:101860. doi: 10.1016/j.cedpsych.2020.101860

[ref67] Ryan R. M. Frederick C. (1997). On energy, personality, and health. Subjective vitality as a dynamic reflection of well-being. J. Pers. 65, 529–565. doi: 10.1111/j.1467-6494.1997.tb00326.x, PMID: 9327588

[ref68] Ryan R. M. Rigby C. S. Przybylski A. (2006). The motivational pull of video games. A self-determination theory approach. Motiv. Emot. 30, 344–360. doi: 10.1007/s11031-006-9051-8

[ref504] Sahu P. (2020). Closure of Universities Due to Coronavirus Disease 2019 (COVID-19): Impact on Education and Mental Health of Students and Academic Staff. Cureus 12:e7541. doi: 10.7759/cureus.754 PMC7198094 32377489

[ref69] Schiefele H. (1974). Lernmotivation Und Motivlernen [Learning Motivation and Motive Learning]. Munich: Ehrenwirth.

[ref70] Schneider M. Preckel F. (2017). Variables associated with achievement in higher education. A systematic review of meta-analyses. Psychol. Bull. 143, 565–600. doi: 10.1037/bul0000098, PMID: 28333495

[ref72] Sheldon K. M. Krieger L. S. (2007). Understanding the negative effects of legal education on law students. A longitudinal test of self-determination theory. Personal. Soc. Psychol. Bull. 33, 883–897. doi: 10.1177/0146167207301014, PMID: 17483395

[ref73] Sheldon K. M. Osin E. N. Gordeeva T. O. Suchkov D. D. Sychev O. A. (2017). Evaluating the dimensionality of self-determination theory’s relative autonomy continuum. Personal. Soc. Psychol. Bull. 43, 1215–1238. doi: 10.1177/0146167217711915, PMID: 28903685

[ref74] Staller N. Großmann N. Eckes A. Wilde M. Müller F. H. Randler C. (2021). Academic self-regulation, chronotype and personality in university students during the remote learning phase due to COVID-19. Front. Educ. 6:797. doi: 10.3389/feduc.2021.681840

[ref75] Taylor G. Jungert T. Mageau G. A. Schattke K. Dedic H. Rosenfield S.. (2014). A self-determination theory approach to predicting school achievement over time. The unique role of intrinsic motivation. Contemp. Educ. Psychol. 39, 342–358. doi: 10.1016/j.cedpsych.2014.08.002

[ref76] Tezci E. Sezer F. Gurgan U. Aktan S. (2017). A study on social support and motivation. Anthropologist 22, 284–292. doi: 10.1080/09720073.2015.11891879

[ref77] Thomas A. E. Müller F. H. Bieg S. (2018). Entwicklung und Validierung der Skalen zur motivationalen regulation beim Lernen im Studium [development and validation of motivational regulation scales in undergraduate learning] (SMR-LS). Diagnostica 64, 145–155. doi: 10.1026/0012-1924/a000201

[ref78] United Nations Educational Scientific, and Cultural Organization (UNESCO) (2020). Education from disruption to recovery. Available at: https://en.unesco.org/covid19/educationresponse (Accessed May 27, 2020).

[ref79] Vallerand R. J. Pelletier L. G. Blais M. R. Briere N. M. Senecal C. Vallieres E. F. (1992). The academic motivation scale. A measure of intrinsic, extrinsic, and amotivation in education. Educ. Psychol. Meas. 52, 1003–1017. doi: 10.1177/0013164492052004025

[ref80] Vandenkerckhove B. Soenens B. van der Kaap-Deeder J. Brenning K. Luyten P. Vansteenkiste M. (2019). The role of weekly need-based experiences and self-criticism in predicting weekly academic (mal)adjustment. Learn. Individ. Differ. 69, 69–83. doi: 10.1016/j.lindif.2018.11.009

[ref81] Vansteenkiste M. Ryan R. M. (2013). On psychological growth and vulnerability. Basic psychological need satisfaction and need frustration as a unifying principle. J. Psychother. Integr. 23, 263–280. doi: 10.1037/a0032359

[ref82] Vansteenkiste M. Ryan R. M. Soenens B. (2020). Basic psychological need theory. Advancements, critical themes, and future directions. Motiv. Emot. 44, 1–31. doi: 10.1007/s11031-019-09818-1

[ref83] Vermote B. Waterschoot J. Morbée S. van der Kaap-Deeder J. Schrooyen C. Soenens B.. (2021). Do psychological needs play a role in times of uncertainty? Associations with well-being during the COVID-19 crisis. J. Happiness Stud. 395:e37. doi: 10.1007/s10902-021-00398-x PMC8081282 33942013

[ref84] Wang C. Hsu H.-C. K. Bonem E. M. Moss J. D. Yu S. Nelson D. B.. (2019). Need satisfaction and need dissatisfaction. A comparative study of online and face-to-face learning contexts. Comp. Human Behav. 95, 114–125. doi: 10.1016/j.chb.2019.01.034

[ref86] Wong R. (2020). When no one can go to school. Does online learning meet students’ basic learning needs? Interact. Learn. Environ. 72, 1–17. doi: 10.1080/10494820.2020.1789672

